# Factors associated with access to condoms and sources of condoms during the COVID-19 pandemic in South Africa

**DOI:** 10.1186/s13690-021-00701-5

**Published:** 2021-10-27

**Authors:** Obasanjo Afolabi Bolarinwa

**Affiliations:** 1 Discipline of Public Health Medicine, School of Nursing and Public Health, University of KwaZulu-Natal, Durban, South Africa; 2 Obaxlove Consult, Lagos, 100009 Nigeria; 3 Department of Global Public Health, School of Allied and Public Health Professions, Canterbury Christ Church University, Canterbury, CT1 1QU UK

**Keywords:** Access, Sources, Condoms, Sexual health, COVID-19, South Africa

## Abstract

**Background:**

Evidence has shown that the prescribed lockdown and physical distancing due to the novel coronavirus disease 2019 (COVID-19) have made accessing essential health care services much more difficult in low-and middle-income countries. Access to contraception is an essential service and should not be denied, even in a global crisis, because of its associated health benefits. Therefore, it is important to maintain timely access to contraception without unnecessary barriers. Hence, this study examines the factors contributing to limited access to condoms and sources of condoms during the COVID-19 pandemic in South Africa.

**Methods:**

This study used the National Income Dynamics Study-Coronavirus Rapid Mobile Survey (NIDS-CRAM) wave 1 survey dataset. The NIDS-CRAM is a nationally representative survey of the National Income Dynamics Survey (NIDS) conducted via telephone interview during COVID-19 in the year 2020. This is the first secondary dataset on COVID-19 conducted by NIDS during pandemic. A total of 5304 respondents were included in the study. Data were analysed using frequencies distribution percentages, chi-square test and multivariable logistic regression analysis.

**Results:**

Almost one-quarter (22.40%) of South Africans could not access condoms, and every 7 in 10 South Africans preferred public source of condoms during the COVID-19 pandemic. Those who were from other population groups [AOR = 0.37; 95% CI = 0.19–0.74] and those who were in the third wealth quintile [AOR = 0.60; 95% CI = 0.38–0.93] had lower odds of having access to condoms while those respondents who were aged 25–34 [AOR = 0.48; 95% CI = 0.27–0.83] and those with a secondary level of education and above [AOR = 0.24; 95% CI = 0.08–0.71] were less likely to prefer public source of condom.

**Conclusions:**

This study concludes that there was limited access to condoms during the COVID-19 pandemic and that the preferred source of condoms was very skewed to public sources in South Africa. Strategic interventions such as community distribution of free condoms to avert obstruction of condom access during the COVID-19 pandemic or any future pandemics should be adopted.

## Background

The highly contagious coronavirus disease 2019 (COVID-19) outbreak has revealed how strikingly unprepared the world is for a pandemic and how easily viruses spread in our interconnected world, which has radically changed social relations in the world [1–3]. The first cases of COVID-19 were declared in Africa in late February and early March 2020 [4]. South Africa (SA) had its first case reported on March 06, 2020 [5]; since then, cases have increased to over 1,170,590 and more than 31,809 deaths have been recorded as of January 10, 2021 [6].

Subsequently, President Cyril Ramaphosa declared a nationwide lockdown on 23 March 2020 to help curb the spread of the COVID-19 in South Africa and encourage health systems to plan for the influx of moderate to severe COVID-19 cases [7, 8]. In addition to the national lockdown, other physical distancing steps such as isolation of persons infected with the COVID-19 and quarantining of anyone who might have been exposed or in contact with an infected individual was also encouraged and implemented [9].

Despite the World Health Organization (WHO) advice to national leaders that COVID-19 preparedness efforts should focus on access to “essential medicines” and healthcare services, to prioritise other health needs of the population whilst the Nation is on lockdown [10], some individuals within households and communities in South Africa were deprived of access to essential medicine or health care services, including sexual and reproductive health services, because they feel obligated to uphold the lockdown and prevent transmission of COVD-19 [11].

The strain that the outbreak imposes on health systems will undoubtedly impact the sexual and reproductive health needs of individuals living in low-and middle-income countries (LMICs) [12–15].

Prior to COVID-19 pandemic, LMICs within sub-Saharan Africa (SSA) and Southern Asia bore the maximum burden of unmet need for modern contraceptives, accounting for 57% of total global unmet needs, of which 39% of these women reside in developing countries [16], while South Africa’s overall unmet need for contraception was 18%, with contraceptive prevalence rate (CPR) for married women at 54, 64% for unmarried women and male condoms use rate of 16% [17].

Guttmacher Institute Authors and other studies estimated that if there were a 10% decline over a year in the use of contraception as a result of limited access because of the ongoing pandemic, an additional of over 48 million women would have an unmet need for contraception worldwide, resulting in more than 15 million additional unintended pregnancies [18–20], which may lead to unsafe abortions and higher extra spending in the future on sexual and reproductive health outcomes as a result of COVID-19 pandemic [21–23].

However, prior to the COVID-19 pandemic outbreak, multiple factors such as poverty, illiteracy, lack of knowledge and awareness about contraceptives, non-availability of contraceptives and socio-demographic inequalities have been linked to low use of contraceptives [16, 24–26], but the most recent is the limited access to sexual and reproductive healthcare services due to COVID-19 outbreak which made access to their choice of contraception limited as well [27, 28]. Limited access to condom use during the COVID-19 outbreak has been previously linked to risky sexual behaviour among nine out of every fifteen adults in Italy [29].

Condom use has been recognised as one of the most effective contraceptive methods of preventing unintended pregnancy and sexually transmitted infections [30, 31]. Given this dual usefulness, condom services or availability at any point in time should not be obstructed. Thus, there is a need to examine factors associated with condoms access and its sources during the ongoing COVID-19 pandemic in South Africa.

The outcome of this study will be useful to South African health authorities in implementing required interventions that will put into consideration factors contributing to limited access to condom use and preferred source of condoms.

## Methods and materials

### Data source and study design

This study used the National Income Dynamics Study-Coronavirus Rapid Mobile Survey (NIDS-CRAM) wave 1 dataset [32]. NIDS-CRAM is a nationally representative survey of the National Income Dynamics Study (NIDS) wave 5 conducted in the year 2017, which involves a sample of South Africans who were then re-interviewed via telephone during the COVID-19 pandemic in South Africa [33]. This is the first secondary dataset on COVID-19 from NIDS conducted during the COVID-19 pandemic [34]. This survey’s primary investigator is the Southern Africa Labour and Development Research Unit (SALDRU), affiliated with the University of Cape Town (UCT). SALDRU is aided by the South Africa Department of Planning, Monitoring and Evaluation [35]. The survey is designed to be nationally representative and remains the best available source of quantitative information on a national scale to assess the health care needs and socio-economic impact of the COVID-19 pandemic in South Africa [36].

### Definition of study variable

#### Outcome variable

The outcome variables were “access to condoms” and “sources of condoms”. Access to condoms was measured by asking the respondents if they have access to condoms (either male or female condoms) during COVID-19 lockdown or not. Those who had access were coded “1” as “yes,” and those who did not have access were coded “0” as “No” [37, 38]. Source of condoms has three variables which were private sources (respondent getting condoms from the private source like private clinic or hospital), public (respondent getting condoms from the public source like public/government clinic or hospital) and other sources which include pharmacy shops, road sellers etc.). These three variables were categorised as “public sources” and “private sources” by adding other sources to private sources [39].

#### Explanatory variables

The explanatory variables were the demographic and economic characteristics of the respondents. Selected demographic and economic characteristics were age, population group, gender and province, employment status, educational level, and respondents’ wealth quintile. The wealth quintile of the respondents was measured using the nation’s wealth quintile categorization (Upper quintile: R52 078 and above, 4th quintile: R23 156 – R52 077, 3rd quintile: R12 781 – R23 155, 2nd quintile: R7 030 – R12 780, and Lower quintile: R7 029 and below) [40, 41].

### Data collection and sampling procedure

The NIDS-CRAM is a computer-assisted telephone interviewing (CATI) first wave survey, conducted during the COVID-19 pandemic in South Africa from May to June 2020. The NIDS-CRAM sample was drawn using a stratified sampling design [32]. The response rate was approximately 40%. The sampling process employed a non-response adjustment by oversampling strata where strata response rates in the initial batches were low. A further 8% of the selected respondents were classified as non-response. The non-response adjustment is then undertaken using the design weight by multiplying the inverse of the conditional probability of those interviewed [42].

Respondents were mainly asked retrospective questions about their circumstances from February to April 2020 using a questionnaire aided by CATI. The NIDS-CRAM constitutes a sample of 7073 individuals drawn from the adult sub-sample of the fifth wave of NIDS conducted in the year 2017. More information about the NIDS-CRAM wave 1 dataset and sample design has been published elsewhere by other authors who used the dataset [36, 43, 44].

Information such as demographic and economic characteristics, access to condoms and sources of condoms during the COVID-19 pandemic in South Africa were the variables extracted from the NIDS-CRAM wave 1 dataset. The de-identified dataset can be accessed upon request at http://www.nids.uct.ac.za, while the redefined dataset used for this study has been deposited to open science framework (OSF) accessible here https://doi:10.17605/OSF.IO/J4XQR.

After eliminating respondents who failed to answer questions related to access to condoms or source of condoms during COVID-19 pandemic in South Africa, a total of 5304 respondents were eligible for this study out of 7073 individuals interviewed during the survey. The eligible respondents were male and female of reproductive age group between the age of 15 to 49. This is because the reproductive health age group is often defined as those between the ages of 15 and 49. These are the age groups assumed to be more sexually active and are majorly in need of sexual and reproductive health services, including condoms [45, 46].

### Statistical analysis

NIDS-CRAM wave 1 dataset was recoded and analyzed using Stata version 16 software. Descriptive statistics were used to summarize data on demographic characteristics, economic characteristics, access to condoms and preferred sources during COVID-19 pandemic in South Africa. Dataset was weighted by applying the recommended weight command to avoid over-sampling, and for non-response adjustment, the outputs were summarized as percentages (%) for both explanatory and outcome variables [32]. Chi-square (χ^2^) was done to check the significant association of the selected demographic and economic variables on access to condoms and sources of condoms, and afterward, multivariable logistic regression test was performed to determine the adjusted likelihood of the explanatory variables on only access to condoms and condom source.. The multivariable logistics regression results were presented in adjusted odds ratio (AOR) with their corresponding 95% confidence intervals (CIs), and AOR less than 0.05 is considered statistically significant. The multicollinearity test, which used the variance inflation factor (VIF), revealed no collinearity among the explanatory variables employed in this study.

### Ethics approval and consent to participate

This study is a secondary analysis of the NIDS-CRAM wave 1 dataset. Ethical approval for NIDS-CRAM was granted by the University of Cape Town (UCT) Commerce Faculty Ethics Committee. In 2017, the NIDS data collectors (Wave 5) conducted a written informed consent process for all participants and only resumed interviews until this procedure had been completed. NIDS-CRAM wave 1 conducted in the year 2020 was drawn from the same population sample of the NIDS wave 5; hence, the participants’ consent was re-validated via telephone interview before proceeding with NIDS-CRAM wave 12,020 questions.

## Results

### Percentage distribution of the outcome and explanatory variables

#### Outcome variables

**Fig. 1:**
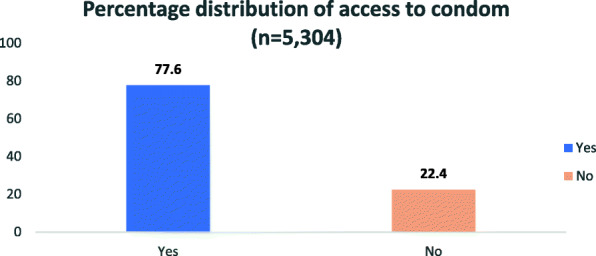
Percentage distribution of access to condom

**Fig. 2:**
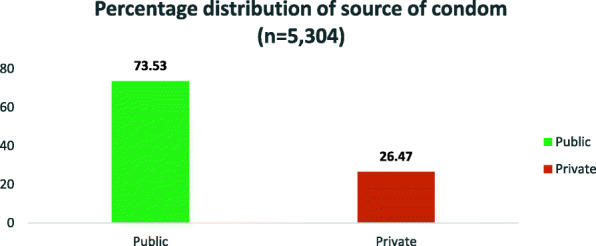
Percentage distribution of source of condom

The percentage distribution of the outcome variables presented in Figs. 1 and 2 below indicated that 22.40% of the respondents were unable to access condoms during COVID-19 pandemic in South Africa, while 7 out of every 10 respondents preferred public sources, which include public hospitals and clinics to get condoms (Figs. 1 & 2).

#### NIDS-CRAM wave 1, 2020 (weighted)

Table 1 below showed the percentage distribution of the explanatory variables included in the study.

**Table 1:** Characteristics distribution of respondent’s access to condom and source of condom

Variable *n* = 5304	Weighted Percentage (%)	Access to condom		*χ2 p*-value	Preferred source		*p*-value (χ^2^)
**Age**		No	Yes	0.79	Private	Public	*p* < 0.05
15–24	22.59	76.48	23.52		18.19	81.81	
25–34	34.37	77.99	22.01		28.94	71.06	
35 & above	43.04	77.88	22.41		29.13	70.87	
**Population group**				*p* < 0.05			*p* < 0.001
African/Black	83.07	75.88	24.12		23.30	76.70	
Coloured	8.80	82.97	17.03		31.45	68.55	
Others	8.14	89.41	10.59		91.31	8.69	
**Sex**				0.38			0.13
Male	48.66	78.39	21.61		29.86	70.14	
Female	51.34	76.83	23.17		23.50	76.50	
**Province**				*p* < 0.001			*p* < 0.05
Western Cape	10.69	83.66	16.34		31.68	68.32	
Eastern Cape	11.42	84.51	15.49		27.81	72.19	
Northern Cape	2.85	77.75	22.25		46.79	53.21	
Free State	5.94	73.32	26.68		22.55	77.45	
KwaZulu-Natal	18.18	62.56	37.44		18.27	81.73	
North West	4.78	81.27	18.73		19.55	80.45	
Gauteng	27.39	82.79	17.21		38.57	61.43	
Mpumalanga	9.00	70.79	29.12		24.20	75.08	
Limpopo	9.76	83.40	16.60		22.01	77.99	
**Dwelling Type**				0.14			*p* < 0.01
A Flat House	78.44	78.16	21.84		29.36	70.64	
Traditional House	7.68	71.32	28.68		11.76	88.24	
Informal settlements & others	13.88	77.96	22.04		20.87	79.13	
**Employment**				0.75			*p* < 0.001
Unemployed	50.42	73.31	22.69		17.32	82.68	
Employed	49.58	77.90	22.10		36.01	63.99	
**Wealth quintile**				*p* < 0.01			*p* < 0.001
Lower quintile	59.37	76.79	23.21		23.60	76.40	
2nd quintile	8.56	77.24	22.76		10.83	89.17	
3rd quintile	3.82	84.94	15.06		18.60	81.40	
4th quintile	8.10	71.34	28.66		14.90	85.10	
Upper quintile	20.16	81.27	18.73		53.31	46.69	
**Educational level**				0.10			*p* < 0.001
No Education & Primary education	6.19	71.66	28.34		7.11	92.89	
Secondary education & above	93.81	78.00	22.00		28.11	71.89	

NIDS-CRAM Wave 1, 2020 (Weighted)

About one-third (34.37%) of the respondents were between the age group 25–34 years. Over 8 in 10 (83.07%) of the respondents were Africans or Black, while the lowest population group was among other population groups such as White, Indian, and Asian with 8.14%. A little above half (51.34%) of the respondents were female, while males involved in the survey were below average (48.66%). Gauteng had the highest respondents, with 27.39%, followed by KwaZulu-Natal 18.18%, while the lowest was among Northern-Cape (2.85%). More than 8 in 10 of the respondents dwell in a House or flat residence. More than half (50.42%) of the respondents interviewed were employed, in the same vein, almost three-fifths (59.37%) of the respondents were in the lower quintile. The majority of the respondents had secondary education and above (93.81%).

#### Multivariable analysis

Table 2 showed the adjusted multivariate regression results of access condoms and preferred source of condoms during the COVID-19 pandemic in South Africa.

**Table 2:** Multivariate logistic regression analysis of factors associated with access to condom and source of condom

Variable	Access to condom		source of condom	
*(n =* 5304*)*	Model I (aOR)	95% CI	Model II (aOR)	95% CI
**Age group**				
15–24	RC		RC	
25–34	0.91	0.70–1.19	0.48**	0.27–0.83
35–45	0.97	0.74–1.28	0.57	0.32–1.03
**Population group**				
African/Black	RC		RC	
Coloured	0.85	0.49–1.45	0.98	0.36–2.73
Others	0.37**	0.19–0.74	0.04***	0.01–0.11
**Gender**				
Male	RC		RC	
Female	1.15	0.94–1.40	1.27	0.80–2.03
**Province**				
Western Cape	RC		RC	
Eastern Cape	0.83	0.46–1.50	0.93	0.25–3.43
Northern Cape	1.31	0.65–2.64	0.34	0.08–1.61
Free State	1.63	0.89–2.99	1.14	0.34–3.79
KwaZulu-Natal	2.90***	1.70–4.95	1.30	0.47–3.55
North-West	1.02	0.53–1.99	1.72	0.46–6.52
Gauteng	1.01	0.59–1.72	0.75	0.26–2.21
Mpumalanga	1.89*	1.08–3.32	1.25	0.41–3.83
Limpopo	0.87	0.47–1.60	0.92	0.28–2.99
**Dwelling Type**				
A Flat House	RC		RC	
Traditional House	0.91	0.65–1.27	1.74	0.86–3.52
Informal House & others	0.95	0.70–1.28	1.02	0.52–2.02
**Employment**				
Unemployed	RC		RC	
Employed	1.15	0.91–1.44	0.66	0.41–1.00
**Wealth quintile**				
Lower quintile	RC		RC	
2nd quintile	0.99	0.71–1.36	2.97**	1.31–6.71
3rd quintile	0.60*	0.38–0.93	1.22	0.34–4.39
4th quintile	1.35	0.99–1.86	1.58	0.72–3.47
Upper quintile	0.90	0.67–1.21	0.39**	0.24–0.66
**Educational level**				
No & Primary education	RC			
Secondary education & above	0.80	0.55–1.16	0.24**	0.08–0.71

NIDS-CRAM Wave 1, 2020 (Weighted)

* = *p* < 0.05; ** = *p* < 0.01; *** = *p* < 0.001

*RC* Reference category; *CI* confidence interval; Model I = Adjusted odds ratio for access to condom; Model II = Adjusted odds ratio for condom preferred source

The associated factors with access to condoms were other population groups (White, Indian & Asian), KwaZulu-Natal and Mpumalanga provinces and 3rd wealth quintile. Other population groups such as White, Indian & Asian [aOR = 0.37; 95% CI = 0.19–0.74] and respondents within 3rd wealth quintile [aOR = 0.60; 95% CI = 0.38–0.93] were less likely to have access to condoms compared to respondents who are Black/African and those in the lower wealth quantile while respondents who reside in KwaZulu-Natal [aOR = 2.90; 95% CI = 1.70–4.95] and Mpumalanga [aOR = 1.89; 95% CI = 1.08–3.32] provinces were more likely to have access to condom during COVID-19 compared to those residing in Western Cape province.

Factors associated with preferred condom sources during the COVID-19 pandemic in South Africa were respondent’s age group, population group, wealth quantile and educational level. Respondents between age 25–34 [aOR = 0.48; 95% CI = 0.27–0.83], other population groups such as white, Indian and Asian [aOR = 0.04; 95% CI = 0.01–0.11], respondents within upper quantile [aOR = 0.39; 95% CI = 0.24–0.66], and those with secondary education and above [aOR = 0.24; 95% CI = 0.08–0.71] were less likely to preferred public source of condom during COVID-19 pandemic compared to those between the age of 15–24 years, respondents who were Black/African, those within lower wealth quantile, and those with no education/primary education while respondents within 2nd wealth quantile [aOR = 2.97; 95% CI = 1.31–6.71] were more likely to a preferred public source of condom compared to respondents within lower wealth quintile.

## Discussion

This study examined factors associated with access to condoms and sources of condoms during the COVID-19 pandemic in South Africa using the first NIDS-CRAM wave 1 dataset conducted during the COVID-19 pandemic. This study acquired and contributed to the existing literature on how limited access to condoms can increase the unmet need for contraception, which may lead to adverse sexual and reproductive health outcomes. The study further expands the scope of the unmet need for contraception by including limited access to condoms during the COVID-19 pandemic and physical distancing in South Africa. In the same vein, the study further contributes to the body of knowledge on preferred source of condoms.

This study is in concordance with the study that concluded that limited access to condom contributes to the unmet need for contraception [47]. The result showed that more than two in every ten South Africans experienced limited access to condoms during the pandemic. The limited access to condoms may be due to the mandatory health precautious pronouncement made by the government to curb the spread of COVID-19 in the country [9, 11]. A study conducted in Indonesia and Kenya on preferred contraception sources showed that most people preferred public/government hospitals to obtain contraception prescriptions [48, 49]. This is in line with the study result in that more than two-thirds of South Africans preferred public/government hospitals to get condoms. This could be one of the reasons why the respondents were unable to access condoms as most public or government hospitals were occupied or overwhelmed due to the influx of COVID-19 patients [50, 51] and may also be due to limited transportation as a result of lockdown/ physical distancing [52–54].

As the access to contraception continues to be a major contributor to high unmet needs in developing countries [16], this study results were significant to respondents’ population group, provinces, and wealth quintile. South Africans who were White, Asian, and Indian population groups and those in the third wealth quintile were less likely to experience limited access to condoms during the COVID-19 pandemic. This is contrary to the studies conducted in South Africa and Ghana prior to the COVID-19 pandemic that respondents residing in the rural area, those married and female were less likely to have access to condoms [55, 56].

The results on access to condoms further showed that respondents residing in KwaZulu-Natal and Mpumalanga provinces were more likely to have access to condoms during the COVID-19 pandemic. This corroborates with the findings of Ntshiqa, Musekiwa [57], who reported that there was geographical variation in access to condoms in South Africa.

Factors associated with preferred sources of condoms were age of respondents, population group, wealth index and educational level. The result shows that respondents between the age of 25–34 were less likely to prefer public sources of condoms. This is in line with a study conducted by Radovich, Dennis [58] that reported that young people prefer private sources.

The variation in preferred source of condom reported in this study, in that White, Indian and Asian population group and upper wealth quantile and those with secondary education level and above were less likely to prefer public source of condom while only respondents in second wealth quintile were more likely to prefer public source of condom was similar to a study conducted in Kenya that reported high variation of choice of condom source [49].

Furthermore, this study results showed similarity in most studies, commentaries and editorial opinions that obstruction in sexual and reproductive services in the ongoing COVID-19 pandemic could lead to a high unmet need for contraception [18, 28, 50] and to the best of my knowledge this is the first paper that holistically employed NIDS-CRAM wave 1 dataset to examine factors associated with access to condom and preferred sources of condoms among South Africans during COVID-19 pandemic.

### Strengths and limitations

The use of secondary datasets has its limitations as some questions of interest to further probe the respondents in terms of retrospective questions were not in which limited the study’s scope the scope of the study. Also, the cross-sectional nature of the dataset restricts causality. Nevertheless, this study’s strength is the use of aboriginal staff for the telephone interview with the help of computer-assisted telephone interviewing during the COVID-19 pandemic despite the restriction and lockdown.

### Practical implications and future studies

This study findings underscore the significance of demographic and socioeconomic characteristics as key indicators associated with access to condoms and the source of condoms among South Africans during the COVID-19 pandemic. Obstructed access to sexual and reproductive needs such as condoms and other services are more likely to lead to adverse sexual and reproductive health outcomes such as unintended pregnancy, abortion, maternal mortality etc. The sustainable development goals (SDGs) target 3.7 aim to achieve universal access to sexual and reproductive health care services, including family planning of choice by 2030 [59]. Deliberate policy and interventions specifically designed for non-obstruction of condom use during a pandemic are required to meet the national commitment and SDGs on reproductive health in South Africa. Future studies should consider using qualitative and longitudinal research designs to explore other factors associated with limited condom use during the COVID-19 pandemic in South Africa and why South Africans preferred public sources of condoms, the results from this study and the future new findings could help in designing appropriate programs to promote condom access during COVID-19 pandemic or any other pandemic in the future.

## Conclusions

This study added to the body of literature that there was limited access to condoms during the COVID-19 pandemic and that the preferred source of condoms was very skewed to public sources in South Africa.

The study concluded that the demographic and economic characteristics of South Africans influenced their adopted sources of condoms and that limited access to condoms was more experienced among the African/Black population groups, those who reside in Mpumalanga and KwaZulu-Natal provinces and those who were in the third quintile of wealth quintile. Policies, strategies, and interventions such as community distribution of free condoms to avert obstruction access to condom demands of South Africans. This will reduce the unmet need for contraception in South Africa and tackle the unequal family planning use coverage.

## Data Availability

The de-identified dataset can be accessed upon request at http:/www.nids.uct.ac.za, while the redefined dataset used for this study has been deposited to open science framework (OSF) accessible here https://doi:10.17605/OSF.IO/J4XQR.

## Abbreviations

COVID-19: Coronavirus disease 2019
SSA: Sub-Saharan Africa
LMICs: Low-and-middle-income countries
NIDS: National income dynamic study
WHO: World health organisation
CPR: Contraceptive prevalence rate
NIDS-CRAM: National income dynamics study-coronavirus rapid mobile survey
SALDRU: Southern Africa labour and development research unit
CATI: Computer-assisted telephone interviewing
OSF: Open science framework
CI: Confidence interval
COR: Unadjusted odds ratio
AOR: Adjusted odds ratio
VIF: Variance inflation factor
SDGs: Sustainable development goals
UCT: University of Cape Town
